# Substituting Soybean Meal With Rapeseed Green Cake: A Holistic Assessment of Growth, Metabolic Response, and Intestinal Status in Juvenile Largemouth Bass (*Micropterus salmoides*)

**DOI:** 10.1155/anu/5621022

**Published:** 2026-05-19

**Authors:** Zetian Xia, Haifeng Mi, Mingchun Ren, Dongyu Huang, Xiaoru Chen, Hualiang Liang, Lu Zhang

**Affiliations:** ^1^ Wuxi Fisheries College, Nanjing Agricultural University, Wuxi, 214081, China, njau.edu.cn; ^2^ Tongwei Agricultural Development Co., Ltd., Key Laboratory of Aquatic Nutrition and Smart Farming, Ministry of Agriculture and Rural Affairs, Aquatic Health and Intelligent Aquaculture Key Laboratory of Sichuan Province, Chengdu, 610093, China, agri.gov.cn; ^3^ Key Laboratory of Integrated Rice-Fish Farming Ecology, Ministry of Agriculture and Rural Affairs, Freshwater Fisheries Research Center, Chinese Academy of Fishery Sciences, Wuxi, 214081, China, cafs.ac.cn

**Keywords:** growth, intestinal health, largemouth bass, nutrient metabolism, rapeseed green cake

## Abstract

This study evaluated rapeseed green cake (RGC) as a partial replacement for soybean meal (SBM) in diets for juvenile largemouth bass (*Micropterus salmoides*; 15.63 ± 0.30 g). Five isonitrogenous (47.64%) and isolipidic (11.9%) diets replaced 0% (RGC0), 25% (RGC25), 50% (RGC50), 75% (RGC75), or 100% (RGC100) of SBM protein with RGC. Fish were fed for 8 weeks. The RGC75 group showed significantly lower WGR and SGR and higher CF, whereas SR, FCR, VSI, and HSI were not affected. However, no significant differences were observed in whole‐body composition. Blood analysis revealed reduced total cholesterol (TC) and high‐density lipoprotein (HDL) at RGC ≥ 50 and decreased total protein (TP) at RGC ≥ 75. Alanine aminotransferase (ALT), aspartate aminotransferase (AST), and lipoprotein lipase (LPL) decreased at RGC100, while glucose (GLU) peaked at RGC75/RGC100. Gene expression indicated suppressed protein synthesis (*mtor*, *rps6k*, and *igf1*) at RGC75 and significantly suppressed lipid synthesis (*pparγ* and *fas* at RGC75/RGC100; *srebp1* at RGC100). Gluconeogenesis (*g6pase*) increased at RGC75/RGC100, while glycolysis (*gk*) decreased at RGC ≥ 50. Intestinal health markers showed elevated glutathione (GSH) and malondialdehyde (MDA) at RGC100 and glutathione peroxidase (GPx) activity at RGC ≥ 50. Gene expression analysis showed upregulation of *nf-κb* and downregulation of *il-10* at RGC levels ≥ 50. Downregulation of *nrf2* and *gpx* occurred at RGC levels ≥ 75. *Il-8* expression increased specifically at the RGC100 level. Villus height was significantly reduced at RGC100, accompanied by downregulated expression of tight junction proteins (*occludin* and *claudin-1*) at RGC ≥ 75; intestinal epithelial detachment occurred at RGC100, while goblet cell density increased at moderate RGC replacement (RGC50) but decreased at RGC100. Considering growth, metabolism, and gut health, RGC50 (50% SBM replacement) was optimal.

## 1. Introduction

The largemouth bass (*Micropterus salmoides*) is characterized by rapid growth, short reproductive cycles, strong environmental adaptability, and high‐quality flesh, making it the third‐most farmed carnivorous fish in China, with an annual production exceeding 938,509 tons in 2024 [1]. As a typical carnivorous fish species, largemouth bass have high dietary protein requirements. Huang et al. [2] reported that the crude protein content in diets for largemouth bass should range from 46% to 51% to meet their elevated nutritional demands for both protein and energy. Soybean meal (SBM) plays a dominant role in aquafeeds due to its balanced amino‐acid profile and high metabolizable energy and therefore serves as a key component in the diets of aquatic animals [3]. However, global SBM price volatility and China’s heavy import dependence (72% reliance on high‐risk commodities) necessitate urgent identification of sustainable protein alternatives. China maintains annual soybean imports near 100 million tons, a volume characterized by high external dependence exceeding 85%. The United States and Brazil supply the vast majority, jointly representing ~85% of this substantial import volume [4]. This dependency exposes livestock and aquaculture sectors to geopolitical and environmental risks, exacerbated by supply chain disruptions [5]. Furthermore, extensive SBM use competes with human food resources, threatening food security [6]. Critically, SBM contains antinutritional factors (ANFs) such as phytate and saponins, which impair nutrient absorption and negatively impact aquatic health [7]. Thus, the reduction and substitution of SBM are imperative.

Aligning with FAO’s “Blue Transformation” initiative for protein source diversification [8], rapeseed—the world’s second‐largest oilseed crop (annual yield: 70 million tons; China: 14.7 million tons) [9]—offers rapeseed cake as a viable SBM substitute due to its abundant supply, low cost, and nutritional value. Notably, rapeseed cake composition varies significantly with variety and processing methods [10, 11]. The rapeseed green cake (RGC) used in this study, characterized by its green hue (resulting from minimal heat exposure), preserves native protein structure with crude protein (35%–42%) and crude fat (12%) [12] retention rates substantially higher than those of heat‐pressed meals (<2% crude fat) [13]. Additionally, RGC exhibits superior protein solubility (70%–90% vs. 35%–52% in heat‐pressed meals) [14] and a high methionine content (0.6%–0.8%) [12], effectively compensating for sulfur‐amino‐acid deficits in plant proteins. Its lipid fraction is rich in unsaturated fatty acids (balanced oleic and linoleic acids), while mineral profiles include high calcium (0.6%) and phosphorus (1.0%–1.2%), though 60%–70% of phosphorus exists as low‐bioavailability phytate phosphorus. RGC is also exceptionally rich in selenium and B vitamins (thiamin and niacin) [15].

Despite successful SBM replacement in livestock feeds using double‐low (00‐type) varieties (glucosinolates < 30 μmol/g; erucic acid < 2%), rapeseed cake application in aquaculture remains limited. Conventional rapeseed meals (RSM) contain glucosinolates (50–100 μmol/g), erucic acid, and phytates [16], which reduce protein digestibility and cause hepatopancreatic damage at high inclusion levels [17]. Evidence from multiple fish species—black carp (*Mylopharyngodon piceus*), gibel carp (*Carassius auratus gibelio*), grass carp (*Ctenopharyngodon idellus*), Atlantic salmon (*Salmo salar*), and rainbow trout (*Oncorhynchus mykiss*)—demonstrates that elevated antinutritional factor (ANF) content in RSM adversely affects immunity, digestion, and general physiology, ultimately leading to growth suppression [11]. Specifically, excessive glucosinolates have been demonstrated to impair liver function and induce associated phenomena such as diminished size of thyroid follicle cells, irregular cellular morphology, nuclear enlargement, and increased mitotic activity [18]. Although double‐low varieties reduce glucosinolates [19], existing research focuses on heat‐processed meals, overlooking RGC’s cold‐processing advantages. RGC’s minimal thermal degradation may enhance bioavailability, yet its effects on carnivorous fish remain systematically unevaluated. It can thus be seen that RGC has great prospects for development in the research on SBM substitution.

Therefore, this study focuses on RGC to address industry challenges, and juvenile largemouth bass were selected as the model species, and five isonitrogenous and isoenergetic diets were formulated to evaluate the feasibility and optimal substitution levels of RGC replacing SBM. The findings will provide a theoretical foundation for sustainable RGC utilization in aquafeeds.

## 2. Materials and Methods

### 2.1. Feeding Experiment Design and Test Diets

Five experimental diets were formulated by replacing 0%, 25%, 50%, 75%, and 100% SBM with 0%, 3.65%, 7.35%, 10.9%, and 14.4% RGC, respectively (designated as RGC0 (control group), RGC25, RGC50, RGC75, and RGC100). All diets contained equal amounts of nitrogen (47.7%) and fat (11.9%). Experimental diets were formulated as detailed in Table 1. All ingredients were first pulverized through an 80‐mesh sieve. The resulting powder was homogenized with fish oil and water, then extruded into 2.5 mm pellets using a TSE65 aquafeed extruder (Beijing, China). Following extrusion, pellets were dried at ambient temperature and stored at −20°C until use.

**Table 1:** Ingredients and proximate composition of experimental diets (% dry matter).

Ingredients (%)	Experimental diets				
	RGC0	RGC25	RGC50	RGC75	RGC100
Fish meal^b^	30.00	30.00	30.00	30.00	30.00
Soybean meal^b^	12.00	9.00	6.00	3.00	0.00
Rapeseed green cake^a^	0.00	3.65	7.35	10.90	14.40
Soy protein concentrate^b^	13.40	13.40	13.40	13.40	13.40
Wheat gluten meal^b^	5.00	5.00	5.00	5.00	5.00
Blood meal^b^	2.00	2.00	2.00	2.00	2.00
Chicken meal^b^	10.00	10.00	10.00	10.00	10.00
Fish oil	3.00	3.00	3.00	3.00	3.00
Soybean oil	4.60	4.40	4.20	4.00	3.80
Wheat flour^b^	8.00	8.00	8.00	8.00	8.00
Cassava starch	5.00	5.00	5.00	5.00	5.00
Microcrystalline cellulose	1.80	1.30	0.80	0.40	0.00
Monocalcium phosphate	2.50	2.50	2.50	2.50	2.50
Premix^c^	2.00	2.00	2.00	2.00	2.00
Choline chloride	0.50	0.50	0.50	0.50	0.50
VC	0.10	0.10	0.10	0.10	0.10
Feed mold inhibitor	0.05	0.05	0.05	0.05	0.05
Antioxidant	0.05	0.05	0.05	0.05	0.05
Analyzed proximate composition (% dry matter)					
Crude protein	47.7	47.7	47.7	47.6	47.5
Crude fat	11.7	11.9	11.4	11.4	11.9

*Note:* All main protein ingredients were provided by Wuxi Bioma Tongwei Biotechnology Co., Ltd. (Wuxi, China) unless otherwise stated.

^a^The proximate composition of the rapeseed green cake (RGC) used in this study was provided by the supplier and included moisture 7.2%, digestible energy 2.88 kcal/g, crude protein 35.7%, crude lipid 8.2%, crude ash 6.4%, crude fiber 11.4%, calcium 0.59%, total phosphorus 0.96%, lysine 1.33%, methionine 0.60%, cysteine 0.82%, and protein solubility 70%–90%.

^b^The crude protein contents of fish meal, chicken meal, soy protein concentrate, soybean meal, blood meal, wheat gluten meal, and wheat flour were 65.80%, 62.00%, 63.00%, 46.00%, 90.00%, 80.00%, and 13.10%, respectively; the crude lipid contents were 9.50%, 9.00%, 4.10%, 4.25%, 0.00%, 2.00%, and 4.00%, respectively.

^c^Premix: The premix consisted of vitamin premix and mineral premix, categorized according to their functional components. Both premixes were purchased from HANOVE Biotechnology Co., Ltd. (Wuxi, China), and their composition is expressed in IU or mg per kg of premix.

### 2.2. Fish Management

The feeding trial was conducted using juvenile largemouth bass reared in outdoor net cages at the Nanquan Base of the Freshwater Fisheries Research Center (Wuxi, China). During the pre‐experimental phase, fish were held in 1 m^3^ net cages for 14 days to acclimate, receiving standard feed. Post‐acclimation, juvenile fish (IBW = 15.63 ± 0.30 g) were evenly allocated to 15 net cages (1 m^3^/cage) with 20 individuals per unit. This setup constituted five experimental dietary groups, each assigned to three replicate cages (*n* = 3). Over the 8‐week experimental period, fish were hand‐fed one of the experimental diets twice daily (07:30 and 17:30) to apparent satiety. Daily feed consumption was recorded to calculate the feed conversion ratio (FCR). Water quality was monitored daily throughout the feeding trial. Water quality was measured using a commercially available aquaculture water‐quality test kit (Sampsistemi Biochemistry and Technology Co., Ltd., Beijing, China). The aquatic environment was maintained with dissolved oxygen levels above 6 mg/L, a near‐neutral pH (7.0–7.8), and a temperature range of 25°C–29°C to ensure optimal conditions.

### 2.3. Sample Collection

At the end of the feeding trial, surviving largemouth bass underwent a 24‐h fast. Subsequently, the collected fish were anesthetized with MS‐222 (100 mg/L) for terminal sampling. Individual body weights were recorded, and total numbers per replicate were tallied to determine final body weight (FBW), weight gain rate (WGR), specific growth rate (SGR), and survival rate (SR). For tissue sampling, three individuals were randomly selected from each replicate net cage. Entire carcasses were preserved at −20°C for subsequent analysis of whole‐body composition. Following rapid ice dissection, samples were vitrified in liquid nitrogen and cryoarchived at −80°C for omics studies. The hepatosomatic index (HSI) and viscerosomatic index (VSI) were calculated from the harvested tissues. In addition, two intestinal tissue samples were collected from each group for histological analysis and fixed in 4% paraformaldehyde. Additionally, blood was collected via sterile syringe puncture of the caudal vein into chilled microcentrifuge tubes. The samples were centrifuged at 4000 rpm for 10 min at 4°C to separate plasma, and the supernatant was collected and aliquoted for subsequent analysis.

### 2.4. Analytical Methods

Proximate analyses (moisture, crude protein, crude lipid, and ash) for the experimental diets and whole fish samples were conducted using AOAC methodologies [20]. Plasma biochemical parameters encompassing total protein (TP), alanine transaminase (ALT), aspartate transaminase (AST), triglycerides (TG), total cholesterol (TC), high‐density lipoprotein (HDL), low‐density lipoprotein (LDL), and glucose (GLU) were quantified using a Mindray BS‐400 automatic biochemical analyzer (Shenzhen, China). Malondialdehyde (MDA) concentration was analyzed as an indicator of lipid peroxidation. The activities or levels of key antioxidant components, including superoxide dismutase (SOD), catalase (CAT), glutathione peroxidase (GPx), total antioxidant capacity (T‐AOC), and glutathione (GSH), were also measured to evaluate intestinal oxidative stress. All corresponding assay kits were obtained from the Jiancheng Bioengineering Institute (Nanjing, China). Prior to the biochemical assays, liver tissues were processed to prepare 10% tissue homogenates. Approximately 0.1 g of liver tissue was weighed and transferred into a centrifuge tube, followed by the addition of ice‐cold physiological saline at a ratio of 1:9 (tissue weight:saline volume). The samples were then mechanically homogenized on ice and centrifuged at 4500 rpm for 15 min at 4°C. The resulting supernatant, free of visible debris, was collected for enzyme activity analyses. Protein concentrations in the homogenates were determined according to the instructions provided with the assay kits. All measurements were subsequently performed following the specific procedures recommended by the manufacturer, and enzyme activities were expressed relative to protein content.

### 2.5. RNA Extraction and Real‐Time PCR Analysis

Total RNA extracted from juvenile largemouth bass liver using Vazyme’s RNA isolation reagent was directly analyzed by one‐step qRT‐PCR (One Step SYBR Green Kit, Vazyme, Nanjing). Reactions were run on a Bio‐Rad CFX96 Touch Real‐Time PCR Detection System (Hercules, CA, USA). Primer pairs designed for amplifying the target genes are listed in Table 2. All primers were designed based on the corresponding nucleotide coding sequences (CDS) or predicted transcript sequences provided in the NCBI database, even when the accession numbers were associated with predicted protein entries (XP_), as these records include their respective nucleotide sequences. No primers were designed directly from protein sequences, and all primer pairs were validated through PCR amplification and melting‐curve analysis to ensure specificity. *Gapdh* was selected as the endogenous control based on its consistent expression profile across all experimental treatments, demonstrating no significant variation among groups. Relative gene expression levels were determined using the standard curve method as described by Shao et al. [22].

**Table 2:** RT‐qPCR primer sequences.

Genes	Forward (5′–3)	Reverse (5′–3′)	Primer source
*gapdh*	ACTGTCACTCCTCCATCTT	CACGGTTGCTGTATCCAA	AZA04761.1
Protein metabolism			
* mtor*	TTTGGAACCAAACCCCGTCA	ATCAGCTCACGGCAGTATCG	XM_038723321.1
* rps6k*	TCCAGAGACTCGTGACACCT	AGCTTGGCATACTCTGAGGC	XM_038713349.1
* igf-1*	CCTCTGCCTGTGTATAATCA	TGTCCGTCTTAGCCATCT	XM_038738328.1
* 4ebp1*	CCAGGATCATCTATGACCGAAAG	TGCAGCGATATTGTTGTTGTTC	XM_038703879.1
Lipid metabolism			
* ppar-α*	AGGCTTCATCACCAGAGA	TCCGCAGCAGATAATAGTAG	MK614719.1
* cpt1*	TTACCGTATGGCTATGACTG	GGCTCCGATAACACCTCT	XP_027141042.1
* ppar-γ*	GAGTTCTCAGTCAAGTTCAAC	AATGTAGCACCGTCTCCT	MK614721.1
* fas*	AGTTGAAGGCTGCTGATG	GCTGTGGATGATGTTGGT	XP_028423094.1
* srebp1*	TCACCTTCCTCTTCCTCTC	TCAGTAGCCACACCAGTAT	AFH35105.1
* acc*	TTACATCGCAGCCAACAG	CTCTCCACCTTCCTCTACA	XP_022609673.1
* fabp1*	CTGGAGACTATTACTGGAGAG	ACACAATGCCACCAAGAG	XP_023250027.1
Glucose metabolism			
* pk*	CACGCAACACTGGCATCATC	TCGAAGCTCTCACATGCCTC	MT431526.1
* gk*	CCCTTGTGGGCAGGAGAAAA	ACAACTGAGTCCTCCTTGCG	XP_023260296.1
* g6pase*	ACACAGCAGCCCTGTTCTAC	CCGTTCACACAGTACGGGAT	XM_038735542.1
* pepck*	GGCAAAACCTGGAAGCAAGG	ATAATGGCGTCGATGGGGAC	MT431525.1
* glut2*	GTGTTTGCTGTGCTGCTCCT	GCTCCGTATCGTCTTTGGG	[21]
Antioxidation and immunity			
* gpx*	ATGGCTCTCATGACTGATCCAAA	GACCAACCAGGAACTTCTCAAA	XM_038697220.1
* cat*	CTATGGCTCTCACACCTTC	TCCTCTACTGGCAGATTCT	MK614708.1
* keap1*	GCACCTAACCGTGGAACTCAA	CCAGTTTTAGCCAGTCATTGTTCC	XM_038728593.1
* nrf2*	AGAGACATTCGCCGTAGA	TCGCAGTAGAGCAATCCT	NM_212855.2
* nf-κb*	AGAAGACGACTCGGGGATGA	GCTTCTGCAGGTTCTGGTCT	XM_038699793.1
* il-8*	GAGGGTACATGTCTGGGGGA	CCTTGAAGGTTTGTTCTTCATCGT	XM_038713529.1
* tnfα*	CTTCGTCTACAGCCAGGCATCG	TTTGGCACACCGACCTCACC	XM_038710731.1
* il-10*	CGGCACAGAAATCCCAGAGC	CAGCAGGCTCACAAAATAAACATCT	XM_038696252.1
Tight junction protein			
* occludin*	GATATGGTGGCAGCTACGGT	TCCTACTGCGGACAGTGTTG	XM_038715419.1
* claudin-1*	CCAGGGAAGGGGAGCAATG	GСТCTТТGААССАGТGСGАС	XM_038713307.1
* zo-1*	ATCTCAGCAGGGATTCGACG	CTTTTGCGGTGGCGTTGG	XM_038701018.1
* akp*	GGTTTTCCGGAACAGCACAC	GGCAGTTTGTGTAGGGCTCT	XM_038715644.1
* amylase*	ATGGGTGTGGCTGGATTCAG	GTCTGGTCTGGGTTGATGGG	XM_038717543.1

Abbreviations: acc, acetyl‐coa carboxylase; akp, alkaline phosphatase; cat, catalase; cpt1, carnitine palmitoyltransferase 1; fabp1, fatty acid‐binding protein liver; fas, fatty acid synthase; gapdh, glycerol‐3‐phosphate dehydrogenase; gk, glucokinase; glut2, glucose transporter 2; gpx, glutathione peroxidase; g6pase, glucose‐6‐phosphatase; igf‐1, insulin‐like growth factor 1; il‐8, interleukin 8; il‐10, interleukin 10; keap1, kelch‐like ech‐associated protein 1; mtor, mechanistic target of rapamycin; nf‐κb, nuclear factor kappa‐light‐chain‐enhancer of activated b cells; nrf2, nuclear factor erythroid 2‐related factor 2; pepck, phosphoenolpyruvate carboxykinase; pk, pyruvate kinase; ppar‐γ, peroxisome proliferator‐activated receptor gamma; ppar‐α, peroxisome proliferator‐activated receptor alpha; rps6k, ribosomal protein s6 kinase; srebp1, sterol regulatory element‐binding protein 1; tnfα, tumor necrosis factor alpha; zo‐1, zonula occludens‐1; 4ebp1, eukaryotic translation initiation factor 4e‐binding protein 1.

### 2.6. Intestinal Histology

The paraformaldehyde‐fixed intestinal tissues were dehydrated, embedded in paraffin, sectioned, and subjected to hematoxylin–eosin (H&E) staining. All procedures and staining were performed by Wuhan Servicebio Technology Co., Ltd. (Wuhan, China). Histological images were captured under a light microscope, and ImageJ software (NIH, USA) was used to measure muscular layer thickness, villus length, crypt depth, and goblet cell density.

### 2.7. Statistical Analysis

Superscript letters indicate significant differences (*p* < 0.05) among groups within each parameter. Statistical analyses were performed with SPSS 26.0. Continuous data are expressed as mean ± SD. Group differences were evaluated by one‐way ANOVA; where significant effects were found, Duncan’s test was applied for multiple comparisons.

## 3. Results

### 3.1. Growth and Feed Efficiency

Growth performance in response to the replacement of RGC with SBM is presented in Table 3. The results indicated that WGR and SGR showed significantly lower values in the RGC75 group compared to the control group (*p* < 0.05). The only parameter exhibiting a significant difference at RGC75 was CF, which increased compared to RGC0 (*p*  < 0.05). For SR, FCR, VSI, and HSI, no statistically significant differences were found between the control and treatment groups (*p*  > 0.05).

**Table 3:** Growth response of largemouth bass to experimental dietary regimens.

Items	RGC0	RGC25	RGC50	RGC75	RGC100
IBW (g)	15.63 ± 0.03	15.63 ± 0.03	15.62 ± 0.04	15.62 ± 0.04	15.62 ± 0.03
FBW (g)	65.75 ± 0.97^a^	63.01 ± 0.82^ab^	64.22 ± 0.32^ab^	59.28 ± 1.53^b^	60.91 ± 3.64^ab^
WGR (%)^1^	320.53 ± 5.62^a^	303.05 ± 4.58^ab^	311.23 ± 2.82^ab^	279.53 ± 8.77^b^	290.00 ± 23.28^ab^
SGR (%/day)^2^	2.31 ± 0.02^a^	2.25 ± 0.02^ab^	2.28 ± 0.01^ab^	2.15 ± 0.04^b^	2.19 ± 0.10^ab^
FCR^3^	1.06 ± 0.03	1.09 ± 0.01	1.04 ± 0.03	1.12 ± 0.05	1.10 ± 0.17
CF (g/cm^3^)^4^	2.19 ± 0.07^b^	2.18 ± 0.05^b^	2.28 ± 0.09^ab^	2.46 ± 0.09^a^	2.34 ± 0.06^ab^
VSI (%)^5^	9.62 ± 0.30^ab^	9.13 ± 0.31^b^	10.05 ± 0.20^ab^	10.17 ± 1.21^a^	10.14 ± 0.33^a^
HSI (%)^6^	2.88 ± 0.22	2.78 ± 0.19	3.34 ± 0.17	3.35 ± 0.21	3.15 ± 0.18

*Note:* Values are mean ± SD. Values in the same row with different superscripts are significantly different (*p*  < 0.05).

^1^WGR (%) = 100 × (final body weight − initial body weight) / initial body weight.

^2^SGR (%) = 100 × ln (final body weight / initial body weight) /days of the experiment.

^3^FCR = feed consumed (g, dry weight) / weight gain (g).

^4^CF (g/cm^3^) = 1000 × (final body weight / final length^3^).

^5^HSI (%) = 100 × liver weight / whole‐body weight.

^6^VSI (%) = 100 × viscera weight / whole‐body weight.

### 3.2. Proximate Body Composition

Proximate analysis was conducted on whole‐body samples obtained from largemouth bass following an 8‐week feeding trial with experimental diets (Table 4). Replacement of SBM with rRGC in the diet did not elicit significant alterations (*p*  > 0.05) in whole‐body moisture, crude protein, crude lipid, or ash content of largemouth bass.

**Table 4:** Whole‐body composition of largemouth bass fed on experimental diets (% dry matter).

Items (%)	RGC0	RGC25	RGC50	RGC75	RGC100
Moisture	69.92 ± 0.20	68.84 ± 0.34	68.95 ± 0.80	69.15 ± 0.71	69.03 ± 0.56
Crude lipid	8.53 ± 0.17	8.61 ± 0.16	8.55 ± 0.36	8.24 ± 0.37	8.36 ± 0.27
Crude protein	16.72 ± 0.62	16.59 ± 0.51	16.78 ± 0.25	16.54 ± 0.93	16.73 ± 0.45
Ash	3.26 ± 0.24	3.41 ± 0.37	3.32 ± 0.16	3.37 ± 0.39	3.26 ± 0.39

*Note:* Values are mean ± SD. Values in the same row with different superscripts are significantly different (*p* < 0.05).

### 3.3. Plasma Parameters

The plasma parameters of largemouth bass fed with experimental diets are presented in Table 5. Significant variations were observed across different dietary groups. Compared with the control group, the TP levels were significantly decreased in the RGC75 and RGC100 groups (*p*  < 0.05), and the TG levels reached the lowest level in the RGC75 group (*p*  < 0.05). ALT and AST levels were significantly reduced in the RGC100 group (*p* < 0.05). Compared with RGC0, TC and HDL were significantly decreased in all groups except RGC25 (*p*  < 0.05); LDL was significantly reduced in RGC100 (*p*  < 0.05); GLU was significantly increased in RGC75 and RGC100 (*p*  < 0.05).

**Table 5:** Plasma parameters of largemouth bass fed on experimental diets.

Items	RGC0	RGC25	RGC50	RGC75	RGC100
TP (g/L)	38.76 ± 3.06^a^	36.45 ± 5.30^ab^	34.48 ± 4.11^ab^	33.33 ± 4.33^b^	32.20 ± 3.05^b^
ALT (U/L)	1.18 ± 0.38^a^	1.11 ± 0.29^a^	1.23 ± 0.52^a^	1.46 ± 0.44^a^	0.58 ± 0.12^b^
AST (U/L)	51.18 ± 17.57^a^	45.33 ± 11.57^ab^	54.98 ± 20.57^a^	57.20 ± 20.04^a^	29.79 ± 10.31^b^
TG (mmol/L)	4.56 ± 0.44^a^	4.08 ± 0.94^ab^	4.37 ± 0.58^ab^	3.67 ± 0.58^b^	3.94 ± 1.01^ab^
TC (mmol/L)	8.65 ± 0.55^a^	8.09 ± 1.24^ab^	7.33 ± 0.69^bc^	7.04 ± 0.70^c^	7.01 ± 0.59^c^
HDL (mmol/L)	4.05 ± 0.28^a^	3.77 ± 0.44^a^	3.33 ± 0.32^b^	3.24 ± 0.26^b^	3.21 ± 0.23^b^
LDL (mmol/L)	1.84 ± 0.16^a^	1.70 ± 0.34^ab^	1.59 ± 0.23^ab^	1.53 ± 0.24^ab^	1.49 ± 0.20^b^
GLU (mmol/L)	12.42 ± 2.49^b^	13.36 ± 0.58^ab^	14.03 ± 0.78^ab^	15.24 ± 1.24^a^	14.67 ± 1.78^a^

*Note:* Values are mean ± SD. Values in the same row with different superscripts are significantly different (*p*  < 0.05).

### 3.4. Protein Metabolism

**Figure 1:**
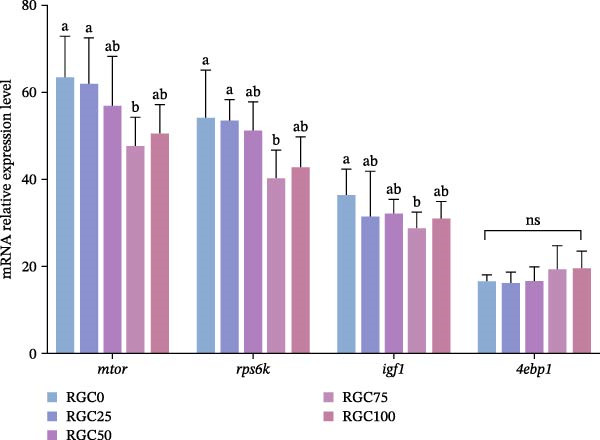
Relative expression of mRNAs for genes related to hepatic protein metabolism in largemouth bass.

Depicted in Figure 1 are the hepatic mRNA expression levels of key genes involved in protein metabolism in largemouth bass. Relative to the RGC0 group, the RGC75 group exhibited significantly lower (*p* < 0.05) mRNA expression of *mtor*, *rps6k*, and *igf1*, representing the minimal levels observed across the dietary treatments. In contrast, hepatic *4ebp1* mRNA levels did not differ significantly (*p*  > 0.05) among the dietary groups.

### 3.5. Lipid Metabolism

**Figure 2:**
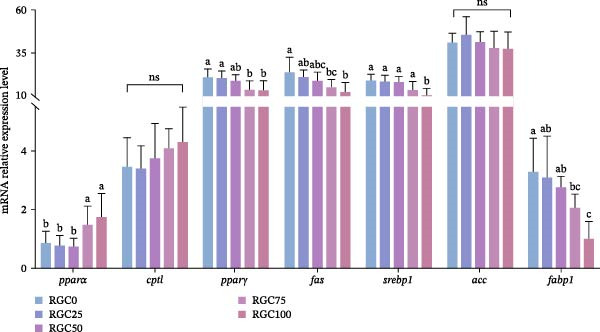
Relative expression of mRNA for genes related to hepatic lipid metabolism in largemouth bass.

The relative hepatic mRNA abundance of genes central to lipid metabolism in largemouth bass is presented in Figure 2. With the increase of RGC substitution in the diet, *ppar-γ*, *fas*, *srebp1*, and *fabp1* showed a decreasing trend. Significant downregulation of *ppar-γ*, *fas*, and *fabp1* was observed in both RGC75 and RGC100 groups relative to RGC0, while *srebp1* expression was only significantly decreased in RGC100 (*p* < 0.05). However, *ppar-α* showed the opposite trend, which reached the highest value in the RGC75 and RGC100 groups compared with the control group (*p* < 0.05). *Cpt1* and *acc* did not show statistically significant differences between the groups (*p* > 0.05).

### 3.6. GLU Metabolism

**Figure 3:**
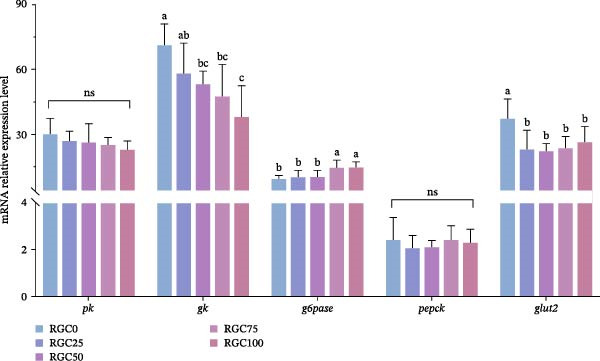
Relative expression of mRNAs for genes related to hepatic glucose metabolism in largemouth bass.

The hepatic transcript abundance of genes involved in GLU metabolism for largemouth bass is detailed in Figure 3. Relative to the control group, both RGC75 and RGC100 dietary treatments induced a significant (*p* < 0.05) upregulation of hepatic g6pase mRNA expression. Conversely, hepatic gk mRNA expression was significantly downregulated (*p* < 0.05) in all treatment groups except RGC25. In addition, the glut2 mRNA levels were all reduced in the RGC replacement groups compared with the RGC0 group (*p* < 0.05), but the difference between pk and pepck was not significant (*p*  > 0.05).

### 3.7. Antioxidation and Immunity in the Intestine

**Figure 4:**
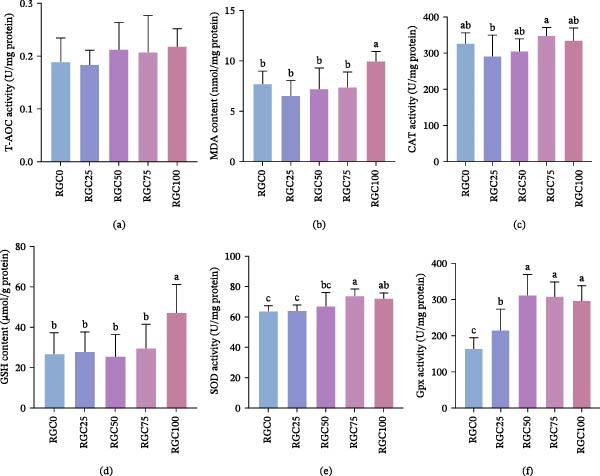
Antioxidant enzyme activities of largemouth bass fed on experimental diets. (a) T‐AOC. (b) MDA. (c) CAT. (d) GSH. (e) SOD. (f) GPx.

As shown in Figure 4, the substitution of RGC for SBM significantly influenced the intestinal antioxidant enzyme activities of largemouth bass. T‐AOC levels (Figure 4a) demonstrated a progressive elevation corresponding to increased replacement levels, although no statistically significant differences were observed among the groups (*p*  > 0.05). In contrast, both MDA (Figure 4b) and GSH (Figure 4d) attained their maximum levels in the RGC100 group, showing marked elevations relative to all other dietary treatments (*p*  < 0.05). Across all dietary treatments and the control, CAT activity (Figure 4c) exhibited no significant alterations (*p*  > 0.05). SOD activity (Figure 4e) reached its peak in the RGC75 group, significantly exceeding levels in the other treatments (*p*  < 0.05). Finally, GPx (Figure 4f) exhibited significantly enhanced activity across all experimental groups compared to the RGC0 (*p*  < 0.05).

**Figure 5:**
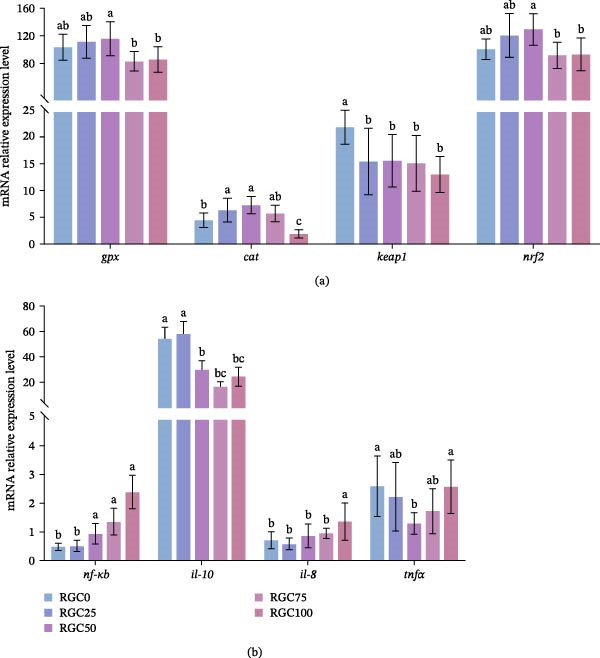
Intestinal antioxidant and immune gene expression in largemouth bass fed different diets. (a) Antioxidant‐related genes. (b) Immune‐related genes.

Figure 5a,b detail the impact of substituting SBM with RGC on the transcription of intestinal antioxidant and immune‐related genes in largemouth bass. As shown in Figure 5a, high RGC inclusion levels (RGC75 and RGC100) led to significant downregulation of both *gpx* and *nrf2* mRNA transcripts compared to lower substitution groups (*p*  < 0.05). *Cat* mRNA expression exhibited a similar trend, reaching its maximum at RGC25 and RGC50 but significantly declining at RGC100 (*p*  < 0.05). Transcript abundance of *keap1* showed a significant downward trend across all RGC substitution groups as the replacement level increased (*p*  < 0.05). As shown in Figure 5b, in groups with ≥50% RGC replacement (RGC50 and RGC100), significant transcriptional alterations were observed for *nf-κb* and *il-10*, characterized by an upregulation of *nf-κb* and a concomitant downregulation of *il-10* (*p*  < 0.05). Furthermore, *il-8* expression was specifically elevated in the RGC100 group compared to other treatments (*p*  < 0.05). Conversely, *tnfα* transcript levels demonstrated an initial decline with increasing substitution, reaching a significantly lower point at RGC50 relative to the control (*p*  < 0.05).

### 3.8. Histopathological Examination

**Figure 6:**
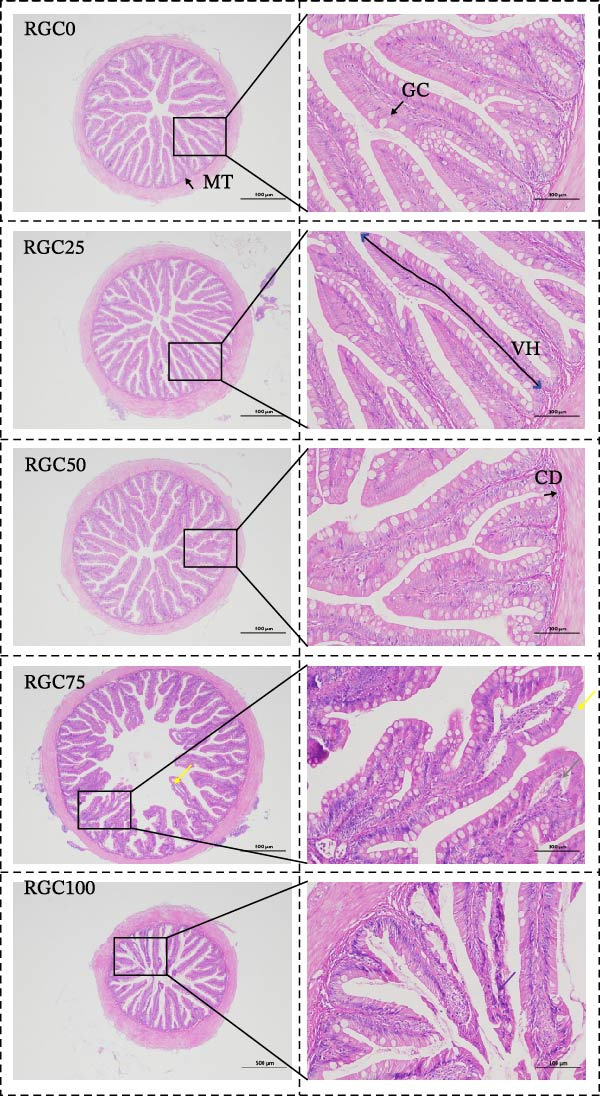
The intestine sections HE staining of the largemouth bass with different levels of RGC (40× and 200×).

As shown in Figure 6, histologic analysis revealed the following results: necrosis and detachment of the intestinal villous mucosal epithelium occurred with increasing substitution of RGC. At 75% substitution, occasional increased cytoplasmic eosinophilia and exposure of the lamina propria were observed. A small amount of intestinal villous epithelium detached from the lamina propria was seen at substitutions up to 100%. As presented in Table 6, villus height was reduced in all largemouth bass relative to the RGC0 (control) group, reaching statistical significance specifically in the RGC100 group (*p*  < 0.05). Notably, significant myofiber hypertrophy was observed with 50% dietary RGC replacement (*p*  < 0.05). In addition, goblet cell density was significantly affected by dietary RGC levels. Goblet cell density was significantly increased in the RGC50 group compared with the RGC0 group (*p*  < 0.05), whereas a marked decrease was observed in the RGC100 group (*p*  < 0.05). No significant differences were detected among the RGC0, RGC25, and RGC75 groups.

**Table 6:** Intestinal histomorphology of *Micropterus salmoides* fed graded RGC levels.

Items	RGC0	RGC25	RGC50	RGC75	RGC100
Villus height (μm)	682.81 ± 89.78^a^	656.39 ± 99.77^ab^	678.02 ± 79.85^ab^	650.83 ± 99.26^ab^	614.54 ± 101.05^b^
Crypt fossa depth (μm)	28.34 ± 5.93	26.86 ± 5.12	25.34 ± 3.00	25.54 ± 5.34	27.88 ± 4.98
Muscle thickness (μm)	168.84 ± 15.24^b^	204.23 ± 29.36^b^	261.48 ± 55.07^a^	214.87 ± 55.75^b^	180.13 ± 31.53^b^
Goblet cell density (cells/mm)	31.43 ± 5.60^b^	36.72 ± 5.46^b^	45.92 ± 8.91^a^	35.47 ± 4.96^b^	24.34 ± 5.77^c^

*Note:* Values are mean ± SD. Values in the same row with different superscripts are significantly different (*p*  < 0.05).

### 3.9. Expression of Genes Related to Intestinal Tight Junction Proteins

**Figure 7:**
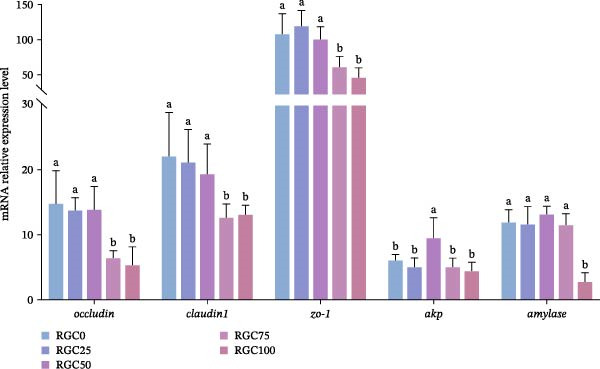
Relative expression of mRNAs for genes related to intestine tight junction proteins in largemouth bass.

In Figure 7, the mRNA expression of *occludin*, *claudin1*, and *zo-1* was significantly lower (*p* < 0.05) in the RGC75 and RGC100 groups compared with the RGC0 group, where the mRNA expression of amylase was significantly lower (*p* < 0.05) than that in the RGC100 group. Meanwhile, the expression of *akp* was significantly (*p* < 0.05) increased in the diet of SBM‐substituted juvenile fish in the RGC50 group.

## 4. Discussion

### 4.1. Growth Performance

This investigation assessed the viability of substituting SBM with rapeseed green meal (RGC) across inclusion levels of 0% to 100% in diets for juvenile largemouth bass. Results indicated that replacement rates reaching 50% (RGC50) did not significantly impair key growth metrics. The absence of growth depression at the 50% replacement level suggests that, at this inclusion rate, the overall dietary protein level and quality still met the nutritional requirements of juvenile largemouth bass and that the dietary load of rapeseed‐derived ANFs remained within a tolerable range. In common carp (*Cyprinus carpio L*.), dietary cold‐pressed rapeseed cake (CPRC) inclusion at 130–200 g/kg did not impair growth, feed utilization, or body composition [23], and even at 330 g/kg substitution, no adverse effects on performance or fillet quality were observed [24]. A broader review on cold‐pressed oilseed cakes also highlighted CPRC as a viable alternative protein ingredient in animal feeds due to its balanced nutrient profile and moderated ANF content [25]. These studies collectively support the present finding that moderate RGC inclusion (≤50%) can be tolerated without detrimental impacts on growth. Similar observations, where moderate RSM inclusion does not impair growth performance until a certain threshold is exceeded, have been reported in tilapia (*Oreochromis niloticus*), hybrid sturgeon (*Acipenser schrenckii ♀* × *Acipenser baerii ♂*) and juvenile largemouth bass [9, 26–28]. Although partial indicators (e.g., WGR, SGR, and FCR) did not continue to deteriorate at a 100% substitution ratio, the comprehensive growth performance remained optimal in the 50% substitution group. At higher inclusion levels (RGC75 and 100), the cumulative load of rapeseed‐derived antinutritional factors may exceed the tolerance threshold of juvenile largemouth bass, thereby constraining growth performance. RSM is known to contain these compounds, which restrict its use in aquafeeds and have been implicated in reduced growth, altered gut health, and decreased digestive capacity in several cultured species [9, 26]. Together with the present growth data, this suggests that the poorer performance observed at RGC75 and 100 is more likely related to reduced efficiency of nutrient utilization than to acute toxicity.

Notably, proximate analysis revealed no significant differences in whole‐body composition (moisture, crude lipid, crude protein, and ash) across all substitution groups, suggesting that the growth depression observed at higher RGC levels was not accompanied by marked changes in gross nutrient deposition. Similar patterns have been observed in other fish species. For example, Li et al. [29] reported that fermented RSM mainly altered serum biochemical and antioxidant indicators in juvenile tilapia, whereas whole‐body composition changed only at the highest inclusion level. Likewise, studies in red sea bream (*Pagrus major*) showed that RSM or fermented RSM affected immune and antioxidant responses without causing marked differences in fillet or whole‐body proximate composition except under excessive replacement [30]. These findings align with the general trend observed in carnivorous fish, where rapeseed‐derived ingredients often induce growth inhibition beyond species‐specific tolerance thresholds [31]. For instance, in rainbow trout (*Oncorhynchus mykiss*), rapeseed protein concentrate (RPC) can replace up to 100% of fish meal without significant negative impact on growth performance [32], whereas in tilapia, even moderate substitution (e.g., 10% RSM) triggers dose‐dependent growth decline [33]. This discrepancy highlights the species‐specific sensitivity to rapeseed‐associated ANFs, such as glucosinolates, tannins, and nonstarch polysaccharides (NSPs), which are known to disrupt digestive enzyme activity and nutrient absorption in fish [18, 34]. Notably, the RGC75 group exhibited a paradoxical increase in CF despite stable FCR, suggesting that reduced growth was not solely due to decreased feed intake. This aligns with studies in tilapia, where RSM beyond 10% substitution elevated CF without altering FCR, attributed to inefficient nutrient utilization rather than anorexia [33]. Unlike growth parameters, SR, HSI, and VSI remained unchanged across groups, indicating that RGC75 did not induce acute toxicity or hepatic stress. Therefore, this study proposes that 7.35% RGC can be utilized in feed formulations to substitute 6.0% SBM, with the maximum allowable substitution level reaching 50%.

### 4.2. Protein Metabolism

Sustained protein synthesis enabled by moderate RGC substitution. RGC inclusion at levels up to 50% SBM replacement supports robust hepatic protein synthesis in largemouth bass. However, beyond this threshold (RGC75 and RGC100), significant reductions in serum TP levels occurred. This decrease correlates with the downregulation of key genes (*mtor*, *rps6k*, and *igf1*) within the mTOR pathway, the central regulator of protein synthesis. This metabolic constraint arises from ANFs in RGC, such as isothiocyanates and tannins, which can inhibit trypsin activity [35] and form complexes with dietary proteins [36], potentially limiting amino‐acid availability.

In most fish studies, increased serum ALT and AST are considered classical indicators of hepatocyte damage and enzyme leakage into the circulation [37]. In the present study, ALT and AST activities were decreased in the RGC100 group. Rather than implying a single outcome, this pattern may have several physiological explanations. First, ALT/AST are key transaminases involved in amino‐acid catabolism and interconversion; therefore, reduced activities may reflect down‐regulated hepatic transamination and overall metabolic depression, which can occur under chronic nutritional stress or ANF exposure [38]. Second, the decreased ALT/AST could indicate limited hepatic enzyme synthesis capacity, consistent with the lowered TP level and suppression of mTOR‐related gene expression observed here. Third, in some nutritional interventions, reduced ALT/AST has also been reported when hepatocyte leakage is not aggravated and may simply suggest no overt liver injury under the current conditions [39]. While the reduced ALT/AST in RGC100 is consistent with an inhibited protein‐metabolic state, the underlying mechanisms remain unclear. Further studies integrating liver histology and additional metabolic biomarkers are needed. Overall, from the standpoint of protein metabolism, RGC can safely replace SBM up to 50% in largemouth bass diets without compromising hepatic protein synthesis, as evidenced by stable TP levels and mTOR‐pathway activity.

### 4.3. Lipid Metabolism

Enhanced lipid utilization through RGC‐driven metabolic shifting, substituting SBM with RGC, promotes a beneficial metabolic shift towards efficient lipid utilization, particularly at the effective 50% replacement level. Serum TC decreased in RGC50, RGC75, and RGC100 groups. This shift was driven molecularly by the pronounced upregulation of *pparα* and its target *cpt1* (promoting mitochondrial β‐oxidation [40]) in RGC75 and RGC100, alongside the downregulation of the lipogenic master regulator *srebp1* and its target *fas* [41]. This metabolic reprograming favors lipid catabolism over synthesis. Serum TG levels reached their lowest point in RGC75, consistent with peak *pparα*/*cpt1* induction. Reduced HDL concentration, potentially impairing reverse cholesterol transport [42], may be compounded by the downregulation of *fabp1*, which limits hepatocyte fatty acid uptake and creates a substrate bottleneck for β‐oxidation despite *pparα* activation [43]. The significant LDL decrease at RGC100, while potentially improving microcirculation [44], requires further investigation regarding hemostasis. The progressive downregulation of the lipid storage regulator *pparγ* further underscores the suppression of lipid anabolism, aligning with reduced adiposity and improved insulin sensitivity in other models [45, 46]. Notably, stable *acc* expression suggests basal lipogenic capacity is preserved, likely to meet essential membrane lipid demands [47]. Molecular analysis revealed that RGC inclusion activates pathways favoring fatty acid oxidation (upregulation of *pparα* and *cpt1*) and moderates fat synthesis (downregulation of *srebp1* and *fas*). This metabolic reprograming enhances energy derivation from lipids, contributing to the efficiency observed with 50% RGC substitution.

### 4.4. GLU Metabolism

Maintained GLU homeostasis with 50% RGC substitution, the largemouth bass effectively maintains GLU balance when RGC replaces up to 50% of dietary SBM. Serum GLU levels remained stable within this optimal substitution range. However, significant hyperglycemia emerged at higher levels (RGC ≥ 75%), likely triggered by ANFs (isothiocyanates and tannins) which may stimulate cortisol‐mediated gluconeogenesis [48]. Molecular profiling revealed hepatic dysregulation wherein ≥75% RGC substitution downregulated *gk* (compromising GLU phosphorylation [49]) and upregulated *g6pase* (accelerating terminal gluconeogenic flux [50]), despite unaltered expression of *pk* (glycolysis) and *pepck* (initial gluconeogenesis). This selective dysregulation established a metabolic imbalance: reduced GLU phosphorylation capacity coupled with enhanced GLU‐6‐phosphate hydrolysis directly potentiated hepatic GLU output. Compounding this, *glut2* suppression across all RGC groups reflected insulin resistance, which may be associated with disrupted PI3K‐AKT signaling and diminished *igf1* expression in RGC75 [17, 51]. Lipid metabolism further amplified dysregulation: elevated *pparα* (RGC75/100) enhanced fatty acid oxidation [52], generating NADPH that altered redox states to broadly inhibit glycolytic enzymes independent of *pk* regulation, while competitively sequestering PGC‐1α to suppress *glut2* transcription [53]. Peak serum GLU concentrations at RGC75 directly correlated with heightened MDA, confirming oxidative stress propagates GLU dysregulation [54]. This underscores the 50% substitution level as a critical threshold for maintaining GLU homeostasis.

### 4.5. Antioxidant and Inflammatory Responses

Boosted antioxidant defenses via RGC polyphenols at optimal inclusion. RGC inclusion, particularly at the beneficial 50% SBM replacement level, actively enhances the endogenous antioxidant system in largemouth bass. In groups receiving ≤50% RGC, a tendency for increased T‐AOC was linked to the induction of key antioxidant genes: *gpx* was significantly upregulated in RGC50, and *cat* showed dose‐dependent activation in RGC25/RGC50. Crucially, *nrf2* activation peaked in RGC50, suggesting polyphenolic components in RGC may beneficially modulate the Nrf2‐Keap1 pathway [55]. While GPx activity remained robustly elevated across all groups, CAT activity showed no change, indicating effective H_2_O_2_ clearance. SOD activity exhibited compensatory upregulation in RGC75/RGC10, which may reflect enhanced superoxide anion scavenging under high substitution. This dynamic dissociation between gene expression and enzyme activity underscores RGC’s multifaceted metabolic interactions. The collapse of oxidative defense at substitution levels ≥75% appeared to represent a critical inflection point. In the RGC100 group, the increase in GSH likely reflected stress‐induced compensatory synthesis. However, the RGC75 and RGC100 groups showed transcriptional suppression of nrf2, which may be associated with reduced nrf2 synthesis caused by lysine‐deficient plant proteins [56]. This impairment precipitated a marked surge in MDA, indicating uncontrolled lipid peroxidation. This oxidative breach may contribute to NF‐κB signaling activation, which may help explain the dose‐dependent *il-8* elevation (peak at RGC100) while suppressing anti‐inflammatory *il-10* from RGC50 onward. Notably, in RGC50, during peak antioxidant defense, IL‐10 suppression and potential NF‐κB activation occurred alongside a reduction in tnfα mRNA, indicating the antioxidant capacity (AOC) was maximally engaged [57]. This shift reflects the dual nature of RGC: antioxidant benefits from polyphenols dominate ≤50%, while ≥75% may trigger phytic acid‐mediated mineral chelation and possible ROS amplification via TLRs/NF‐κB, exceeding endogenous protective capacity [58]. The 50% level represents the optimal window for harnessing RGC’s antioxidant potential.

### 4.6. Intestinal Structural Adaptation and Integrity Limits

Preserved intestinal structure and function with 50% RGC replacement. Carnivorous fish such as largemouth bass generally exhibit a limited tolerance for high plant‐protein substitution because excessive dietary fiber and ANFs can impair digestion and raise oxidative–inflammatory pressure on the intestinal mucosa, leading to villus injury and epithelial instability [39]. Within this adaptive framework, our results indicate that the intestine can accommodate moderate RGC inclusion and maintain structural integrity at ~50% SBM replacement. Such preservation is best interpreted as a compensatory adaptation, in which intestinal remodeling and coordinated protective signaling help sustain barrier homeostasis under a manageable dietary challenge.

In this context, the goblet cell response provides an additional barrier‐level indicator of intestinal adaptation. Goblet cells contribute to mucosal protection primarily through regulated mucus secretion, forming a physical and biochemical interface that reduces luminal irritation and supports epithelial homeostasis. The increased goblet cell density observed at 50% SBM replacement is consistent with an adaptive reinforcement of the mucus barrier under moderate dietary challenge [59].

However, when RGC replacement increased to ≥75%, the dietary fiber/ANF burden likely exceeded the intestinal adaptive capacity, shifting the response from compensation to pathology. High plant‐protein diets have been reported to cause mechanical irritation and villus‐level damage, including mucosal erosion and epithelial sloughing, in fish species exposed to excessive plant‐protein inputs [60]. 59This interpretation is further supported by evidence that plant‐protein‐associated oxidative stress can promote lipid peroxidation and amplify inflammatory injury in largemouth bass when substitution levels are excessive [39]. In largemouth bass, intestinal oxidative stress and inflammation are also closely linked to epithelial cell loss and apoptosis under sustained nutritional stress [61]. Notably, the significant decline in goblet cell density at the highest RGC inclusion level suggests that mucus‐barrier capacity may become insufficient once the tolerance threshold is exceeded, potentially aggravating epithelial vulnerability under sustained oxidative–inflammatory pressure [21]. Therefore, the dose‐dependent villus shortening and epithelial exfoliation observed at high RGC inclusion represent a typical “threshold‐crossing” impairment pattern in carnivorous fish fed excessive plant‐protein diets [62]. Taken together, these findings support 50% SBM replacement by RGC as a practical upper level that preserves intestinal integrity, whereas ≥75% substitution crosses the tolerance threshold and risks progressive barrier injury in largemouth bass, consistent with previous tolerance‐limit observations in plant‐protein substitution studies [62].

### 4.7. Gut Barrier and Digestive Function

Adaptive gut function support at moderate RGC inclusion, RGC substitution stimulates beneficial adaptive responses in gut function up to the 50% SBM replacement level. Notably, the RGC50 group exhibited significant upregulation of *akp* gene expression, representing an adaptive response to dietary challenge and aiding in maintaining nutrient hydrolysis capacity [63]. However, high‐level substitution (≥75% RGC) induced a synergistic downregulation of critical tight junction proteins (*occludin*, *claudin-1*, and *zo-1*). This barrier disruption may involve multiple pathways: TNFα has been suggested to suppress *occludin* via miR‐122a [64], IL‐10 deficiency may reduce anti‐inflammatory protection [65], and MDA‐induced oxidative damage may disrupt ZO‐1/actin connections [66]. The barrier disruption initiated a vicious cycle: impaired nutrient absorption led to growth performance decline, while increased pathogen susceptibility exacerbated secondary infection risks [67]. Digestive function also declined severely at RGC100: phytic acid may inhibit AKP activity through chelation of Zn^2+^ [68–70], while *amylase* gene expression and activity were suppressed, possibly due to zinc deficiency and energy conservation [71]. The transition from adaptive upregulation of AKP at RGC50 to comprehensive suppression of barrier function and digestive enzymes at RGC100 defines the functional limits of RGC substitution, emphasizing the importance of adhering to the ≤50% inclusion level to preserve gut health and nutrient assimilation.

## 5. Conclusion

In conclusion, RGC successfully replaces up to 50% of dietary SBM in largemouth bass feeds, maintaining optimal growth, nutrient retention, and metabolic health. This substitution leverages RGC’s cost‐effectiveness while enhancing antioxidant capacity.

## Author Contributions

**Zetian Xia**: investigation, data curation, writing – original draft. **Haifeng Mi**: supervision, resources. **Mingchun Ren**: writing – review and editing, project administration, funding acquisition. **Dongyu Huang**: investigation, data curation. **Xiaoru Chen**: investigation, project administration. **Hualiang Liang**: data curation, project administration. **Lu Zhang**: investigation, project administration.

## Funding

This study was financially supported by the National Key R & D Program of China (Grant 2024YFD2402005), the earmarked fund for CARS (Grant CARS‐46).

## Ethics Statement

The study was conducted according to the Management Rule of Laboratory Animals (Chinese Order Number 676 of the State Council, revised 1 March 2017). The study was approved by the Laboratory Animal Ethics Committee of the Freshwater Fisheries Research Center (LAECFFRC‐2024–07–10).

## Conflicts of Interest

The authors declare no conflicts of interest.

## Data Availability

The authors confirm that the data supporting the findings of this study are available within the manuscript, tables, and figures. Data are available from the corresponding author upon reasonable request.
